# Correlation of *HAMP* gene polymorphisms and expression with the susceptibility and length of hospital stays in Taiwanese children with Kawasaki disease

**DOI:** 10.18632/oncotarget.17700

**Published:** 2017-05-08

**Authors:** Ying-Hsien Huang, Kuender D. Yang, Yu-Wen Hsu, Hsing-Fang Lu, Henry Sung-Ching Wong, Hong-Ren Yu, Hsing-Chun Kuo, Fu-Chen Huang, Mao-Hung Lo, Kai-Sheng Hsieh, Su-Fen Chen, Wei-Chiao Chang, Ho-Chang Kuo

**Affiliations:** ^1^ Department of Pediatrics and Kawasaki Disease Center, Kaohsiung Chang Gung Memorial Hospital and Chang Gung University College of Medicine, Kaohsiung, Taiwan; ^2^ Institute of Biomedical Sciences, Mackay Medical College, New Taipei City, Department of Pediatrics, Mackay Memorial Hospital, Taipei, and Institute of Clinical Medicine, National Yang Ming University, Taipei, Taiwan; ^3^ The Ph.D. Program for Translational Medicine, College of Medical Science and Technology, Taipei Medical University and Academia Sinica, Taipei, Taiwan; ^4^ Department of Clinical Pharmacy, Taipei Medical University, Taipei, Taiwan; ^5^ Department of Pharmacy, Taipei Medical University-Shuang Ho Hospital, Taipei, Taiwan; ^6^ Master's Program for Clinical Pharmacogenomics and Pharmacoproteomics, School of Pharmacy, Taipei Medical University, Taipei, Taiwan; ^7^ Department of Nursing, Chang Gung University of Science and Technology, Chiayi, Taiwan; ^8^ Research Center for Industry of Human Ecology and Research Center for Chinese Herbal Medicine, College of Human Ecology, Chang Gung University of Science and Technology, Taoyuan, Taiwan; ^9^ Chronic Diseases and Health Promotion Research Center, CGUST, Chiayi, Taiwan; ^10^ Center for Biomarkers and Biotech Drugs, Kaohsiung Medical University, Kaohsiung, Taiwan; ^11^ Department of Pharmacy, St Vincent Medical Center, Los Angeles, California, USA

**Keywords:** hepcidin, genetic polymorphism, kawasaki disease

## Abstract

Kawasaki disease (KD) is a form of systemic vasculitis. Regarding its pathogenesis, *HAMP* gene encoding hepcidin, which is significant for iron metabolism, has a vital function. In this study, we recruited a total of 381 KD patients for genotyping. Data from 997 subjects (500 subjects from cohort 1; 497 subjects from cohort 2) were used for analysis. Using TaqMan allelic discrimination, we determined five tag SNPs (rs916145, rs10421768, rs3817623, rs7251432, and rs2293689). Treatment outcome data related to such clinical phenotypes as coronary artery lesions (CAL), coronary artery aneurysms (CAA), and intravenous immunoglobulin (IVIG) effects were also collected. Furthermore, we measured plasma hepcidin levels with an enzyme-linked immunosorbent assay. We found that *HAMP* gene polymorphism (rs7251432, and rs2293689) was significantly correlated with KD risk and that plasma hepcidin levels both before and after IVIG treatment had a significantly positive correlation with length of hospital stays (R = 0.217, *p* = 0.046 and R = 0.381, *p* < 0.0001, respectively). In contrast, plasma hepcidin levels has a negative correlation with KD patients’ albumin levels (R = −0.27, *p* < 0.001) prior to IVIG treatment. This study's findings indicate that HAMP might have a role in the disease susceptibility, as well as its expressions correlated length of hospital stays, and albumin levels in Taiwanese children with KD.

## INTRODUCTION

A form of acute vasculitis, KD (Kawasaki disease) affects various systems, most often in children under the age of five years old [1]. KD affects the vascular system in both small and medium-sized blood vessels and especially in coronary arteries [2]. As a result, nearly 20% of children who do not receive treatment suffer a coronary artery aneurysm [3]. The most serious complication of KD is the development of coronary artery lesions (CAL), including myocardial infarctions, coronary artery fistula formations [4], coronary artery dilatations, and coronary artery aneurysms (CAA) [5].

In addition to the diagnostic criteria by the American Heart Association and the American Academy of Pediatrics, such nonspecific clinical symptoms as uveitis, aseptic meningitis, gallbladder hydrops, urethritis, arthralgia, arthritis, hypoalbuminemia, liver function impairment anemia, and heart failure have also been found in KD sufferers [6], with anemia being the most commonly found [7–10]. Hepcidin, a protein for iron regulation, reduces iron level by inhibiting intestinal iron absorption and keeping iron storage in macrophages, leading to impaired hemoglobin synthesis [11]. It is essential not only for iron metabolism but also for the pathogenesis of inflammation anemia [11]. Unusually high levels of hepcidin have been found in anemia in various inflammatory disorders, including autoimmune diseases [12, 13], infections [14, 15], critical illnesses [16, 17], and obesity [18]. In our previous study, we also found that abnormally high levels of hepcidin could impair iron metabolism and subsequently correlate with reduced hemoglobin levels in KD patients [19, 20]. Furthermore, in that study, the changes in hepcidin levels after intravenous immunoglobulin (IVIG) treatment were correlated with CAL and IVIG resistance in KD patients [19]. An increasing amount of evidence has shown that iron levels are associated with coronary artery disease [21, 22] and vasculitis [23, 24]. Moreover, as KD occurs more commonly in Asian populations than European populations [2], we also reported that the C allele of rs916145 in the *HAMP* promoter area has a higher frequency of developing biliary atresia, which is consistent with the low incidence of biliary atresia in European populations [25]. However, no correlation studies between *HAMP* and KD have yet been reported. In this study, we aim to determine the role of *HAMP* in children's susceptibility to KD, CAL formation, and CAA, as well as IVIG treatment responses, lengths of hospital stays, and albumin levels.

## RESULTS

### Basal characteristics of KD patients

The KD patients’ basal characteristics for genotyping and plasma hepcidin assay are summarized in Tables 1 and 2, respectively. This study was consisted of 381 KD patients and 997 controls (500 subjects data from cohort 1; 497 subjects data from cohort 2). Male subjects accounted for 66.8% of KD patients and 56% of healthy subjects. A total of 49 (12.9%) KD patients did not respond to their initial IVIG treatment, 64 (16.8%) KD patients developed CAL, and 16 (4.2%) patients developed CAA. The subjects’ *HAMP* genotype distribution agreed with the Hardy-Weinberg equilibrium (Supplementary Table 1).

**Table 1:** Basal characteristics of patients with Kawasaki disease

Characteristics	Patients with KD
	*N* = 381
Male gender, No. (%)	247 (66.8%)
Mean (SD) age (years)	1.7 ± 0.08
Age range (years)	0–11
CAL formation	64 (16.8%)
Aneurysm formation	16 (4.2%)
IVIG resistance	49 (12.9%)

CAL: coronary artery lesions; IVIG: intravenous immunoglobulin; SD: standard deviation.

**Table 2:** Basal characteristics of 85 Kawasaki disease patients for hepcidin assay

Characteristics	pre-IVIG		post-IVIG < 3 days	*p*-value
	*N* = 85			
Mean (SEM), age (m)	21.6 ± 1.8			
Gender (male: female)	53:32			
CAL formation	25 (29%)			
IVIG resistance		7 (8%)		
WBC (1000/uL)	13.8 ± 0.5		10.6 ± 0.5	< 0.0001
CRP (mg/L)	87.2 ± 6.6		46.6 ± 6	< 0.0001
Albumin (g/dL)	3.1 ± 0.06		2.8 ± 0.01	0.007
GOT (U/L)	62.1 ± 7.3		43.4 ± 9.4	0.103
GPT (U/L)	77.1 ± 9.2		42.3 ± 9.4	0.007

CAL: coronary artery lesions; IVIG: intravenous immunoglobulin; CRP: C-reactive protein; GOT: Glutamic Oxaloacetic Transaminase; GPT: Glutamic Pyruvic Transaminase.

### Association study of *HAMP* gene polymorphisms for susceptibility to KD

We investigated the correlations between HAMP genetic polymorphisms and susceptibility for KD. As shown in Table 3, rs2293689 of HAMP significantly correlated with the risk of KD under the allelic models (*p* = 0.0410). Application of second cohort, another polymorphism, rs7251432, was identified to associate with the susceptibility for KD under the recessive model (*p* = 0.0315). (Table 3).

**Table 3:** Genotype and allele frequencies of the *HAMP* gene in controls and patients with Kawasaki disease

	Genotype	Case (%) (*n* =381)	Control cohort 1 (%) (*n*= 497)	Control cohort 2 (%) (*n* =500)	Cohort 1 Recessive *P* Value	Allelic *P* Value	Cohort 2 Recessive *P* Value	Allelic *P* Value
rs916145	CC	46 (13.1)	60 (12.2)	71 (14.2)	0.7108	0.6652	0.6481	0.4768
	CG	158 (45.0)	219 (44.7)	231 (46.2)				
	GG	147 (41.9)	211 (43.1)	198 (39.6)				
rs10421768	GG	0 (0.0)	0 (0.0)	0 (0.0)	1.0000	0.3270	1.000	0.4533
	AG	3 (0.8)	8 (1.6)	7 (1.4)				
	AA	354 (99.2)	489 (98.4)	493 (98.6)				
rs3817623	TT	2 (0.6)	2 (0.5)	1 (0.2)	0.8828	0.3129	03745	0.9983
	GT	30 (8.5)	45 (11.0)	46 (9.2)				
	GG	322 (90.9)	363 (88.5)	453 (90.6)				
rs7251432	GG	67 (18.8)	77 (16.1)	67 (13.4)	0.3120	0.1672	0.0315*	0.0939
	AG	163 (45.8)	212 (44.5)	243 (48.6)				
	AA	126 (35.4)	188 (39.4)	190 (38.0)				
rs2293689	TT	2 (0.6)	4 (1.3)	2 (0.4)	0.3333	0.0410*	0.7366	0.8016
	CT	44 (12.3)	54 (17.0)	60 (12.0)				
	CC	312 (87.1)	260 (81.7)	438 (87.6)				

*Significant (*P* < 0.05) values are in bold.

### Lack of correlation between *HAMP* polymorphisms and CAL formation, IVIG treatment, and aneurysm formation in KD patients

We also tested whether the *HAMP* polymorphisms correlated with the clinical phenotypes, but none of the polymorphisms had an association with CAL, CAA, or response to IVIG treatment (Tables 4, 5, and 6).

**Table 4:** Genotype and allele frequencies of *HAMP* gene in Kawasaki disease patients with or without coronary artery lesion formation

	Genotype	CAL 2 (%) (*n* =64)	Without (%) (*n* =310)	Allele	CAL 2 (%) (*n* =64)	Without (%) (*n* =310)	Genotype *p*-value	Dominant *p*-value	Recessive *p*-value	Allelic *p*-value
rs916145	CC	10 (18.2)	36 (12.3)	C	47 (42.7)	202 (34.6)	0.2696	0.1501	0.2403	0.1026
	CG	27 (49.1)	130 (44.5)	G	64 (57.3)	382 (65.4)				
	GG	18 (32.7)	126 (43.2)							
rs10421768	GG	0 (0.0)	0 (0.0)	G	1 (0.9)	2 (0.3)	-	0.4275	-	0.4285
	AG	1 (1.7)	2 (0.7)	A	115 (99.1)	588 (99.7)				
	AA	57 (98.3)	293 (99.3)							
rs3817623	TT	0 (0.0)	2 (0.7)	T	5 (4.3)	28 (4.8)	0.8158	0.9447	0.5273	0.8222
	GT	5 (8.6)	24 (8.2)	G	111 (95.7)	556 (95.2)				
	GG	53 (91.4)	266 (91.1)							
rs7251432	GG	8 (13.6)	56 (19.1)	G	44 (37.3)	247 (42.2)	0.5787	0.5414	0.3130	0.3278
	AG	28 (47.4)	135 (46.1)	A	74 (62.7)	339 (57.8)				
	AA	23 (39.0)	102 (34.8)							
rs2293689	TT	0 (0.0)	2 (0.7)	T	4 (3.3)	43 (7.3)	0.2682	0.1127	0.5176	0.1013
	CT	4 (6.6)	39 (13.3)	C	118 (96.7)	543 (92.7)				
	CC	57 (93.4)	252 (86.0)							

**Table 5:** Genotype and allele frequencies of the *HAMP* gene in Kawasaki disease patients with aneurysm or without aneurysm

	Genotype	Aneurysm (%) (*n* = 16)	Without (%) (*n* = 362)	Allele	Aneurysm (%) (*n* = 16)	Without (%) (*n* = 362)	Genotype *p*-value	Dominant *p*-value	Recessive *p*-value	Allelic *p*-value
rs916145	CC	1 (7.1)	45 (13.4)	C	10 (35.7)	240 (35.6)	0.6052	0.6332	0.4999	0.9908
	CG	8 (57.1)	150 (44.5)	G	18 (64.3)	434 (64.4)				
	GG	5 (35.7)	142 (42.1)							
rs10421768	GG	0 (0.0)	0 (0.0)	G	0 (0.0)	3 (0.4)	-	0.7253	-	0.7258
	AG	0 (0.0)	3 (0.9)	A	28 (100.0)	683 (99.6)				
	AA	14 (100.0)	340 (99.1)							
rs3817623	TT	0 (0.0)	2 (0.6)	T	2 (6.7)	32 (4.7)	0.7571	0.5534	0.7655	0.6255
	GT	2 (13.3)	28 (8.3)	G	28 (93.3)	646 (95.3)				
	GG	13 (86.7)	309 (91.1)							
rs7251432	GG	3 (20.0)	64 (18.8)	G	14 (46.7)	283 (41.5)	0.7621	0.4702	0.9049	0.5740
	AG	8 (53.3)	155 (45.4)	A	16 (53.3)	399 (58.5)				
	AA	4 (26.7)	122 (35.8)							
rs2293689	TT	0 (0.0)	2 (0.6)	T	0 (0.0)	48 (7.0)	0.2909	0.1161	0.7590	0.1208
	CT	0 (0.0)	44 (12.9)	C	32 (100.0)	636 (93.0)				
	CC	16 (100.0)	296 (86.5)							

**Table 6:** Genotype and allele frequencies of the *HAMP* gene in Kawasaki disease patients that respond or do not respond to intravenous immunoglobulin treatment

	Genotype	Resistant (%) (*n* = 49)	Responsive (%) (*n* = 326)	Allele	Resistant (%) (*n* = 49)	Responsive (%) (*n* = 326)	Genotype *p*-value	Dominant *p*-value	Recessive *p*-value	Allelic *p*-value
rs916145	CC	5 (10.6)	40 (13.2)	C	36 (38.3)	212 (35.1)	0.3306	0.2444	0.6199	0.5467
	CG	26 (55.3)	132 (43.7)	G	58 (61.7)	392 (64.9)				
	GG	16 (34.0)	130 (43.1)							
rs10421768	GG	0 (0.0)	0 (0.0)	G	0 (0.0)	3 (0.5)	-	0.5068	-	0.5077
	AG	0 (0.0)	3 (1.0)	A	45 (100.0)	615 (99.5)				
	AA	45 (100.0)	306 (99.0)							
rs3817623	TT	0 (0.0)	2 (0.7)	T	6 (6.4)	28 (4.6)	0.4642	0.3464	0.5777	0.4504
	GT	6 (12.8)	24 (7.9)	G	88 (93.6)	582 (95.4)				
	GG	41 (87.2)	279 (91.5)							
rs7251432	GG	7 (15.2)	60 (19.5)	G	37 (40.2)	258 (41.9)	0.7292	0.9020	0.4911	0.7624
	AG	23 (50.0)	138 (44.8)	A	55 (59.8)	358 (58.1)				
	AA	16 (34.8)	110 (35.7)							
rs2293689	TT	0 (0.0)	2 (0.7)	T	7 (7.3)	41 (6.7)	0.7618	0.7184	0.5749	0.8236
	CT	7 (14.6)	37 (12.0)	C	89 (92.7)	573 (93.3)				
	CC	41 (85.4)	268 (87.3)							

### Correlation between length of hospital stays and hepcidin levels in KD patients

**Figure 1:**
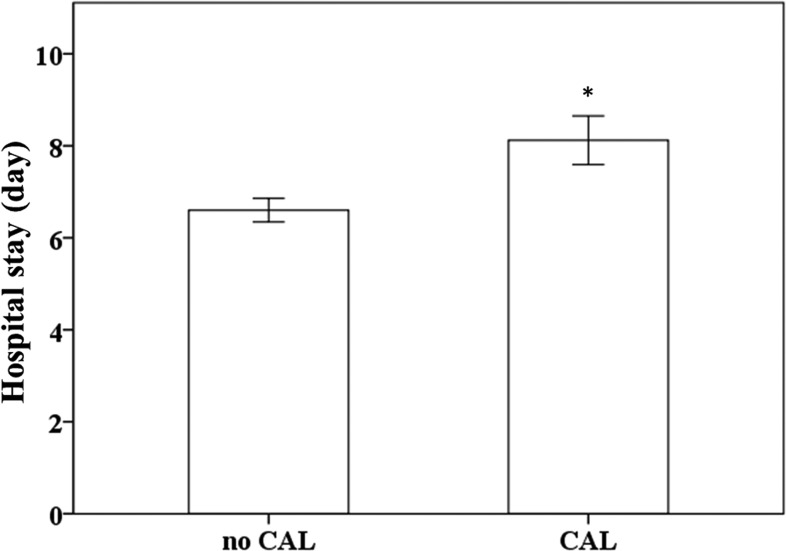
Comparison of KD patients’ length of hospital stays Data are presented as mean ± standard error. * indicates *p* < 0.05 between groups. KD indicates Kawasaki disease, CAL indicates coronary artery lesions.

**Figure 2:**
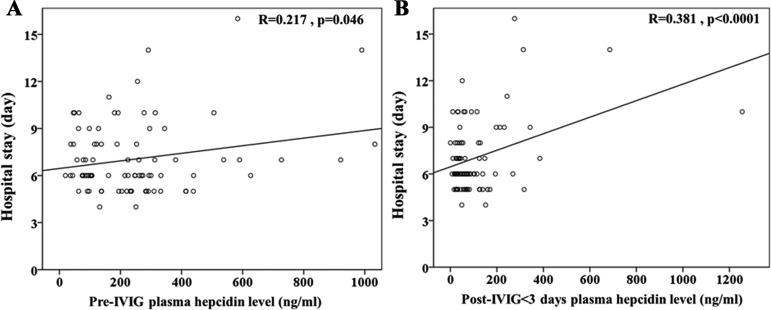
Univariate analysis illustrates that plasma hepcidin levels both (**A**) before and (**B**) after IVIG treatment were positively correlated with patients’ length of hospital stays (*R* = 0.217, *p* = 0.046, and *R* = 0.381, *p* < 0.0001, respectively).

In a prior study, we showed that the changes in hepcidin levels were related to the development of CAL and the response for IVIG treatment in KD patients [19]. In this study, we showed that longer hospital stays were observed in KD patients with CAL than in those without CAL (*p* = 0.005) (Figure 1). Furthermore, we confirmed using linear regression modeling that plasma hepcidin levels both before and after receiving IVIG treatment were positively correlated with patients’ length of hospital stays (*R* = 0.217, *p* = 0.046 and *R* = 0.381, *p <* 0.0001, respectively), as shown in Figure 2.

### Correlation between AST, ALT, albumin, and hepcidin levels in KD patients

**Figure 3:**
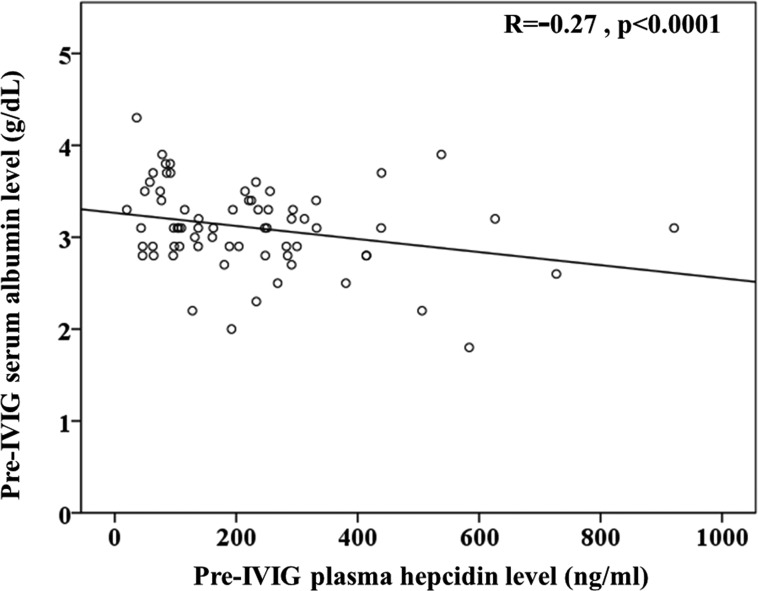
Univariate analysis illustrates that plasma hepcidin was negatively correlated with (c) albumin (R = −0.27, p < 0.001) prior to IVIG treatment in KD patients.

KD patients often present with hepatitis and jaundice [26], and since hepatocytes are the major producer of hepcidin, that could be directly related to the reason for modified hepcidin expression in KD patients. Furthermore, lower albumin levels were correlated with a resistance to intravenous immunoglobulin treatment and CAL formation [27–29]. We found that plasma hepcidin levels were negatively correlated with albumin (R = −0.27, *p <* 0.001) prior to IVIG treatment in KD patients (Figure 3).

## DISCUSSION

We reported herein that *HAMP* correlated with KD patients’ disease susceptibility and length of hospital stays. Furthermore, rs2293689 of *HAMP* is an important marker with a significant correlation with KD risk. Plasma hepcidin levels in the KD patients both before and after IVIG treatment were positively correlated with patients’ length of hospital stays. In contrast, plasma hepcidin was negatively correlated with albumin before KD patients received IVIG treatment.

While there are many elevated inflammatory mediators in KD patients’ peripheral blood, no particular biomarker can be used to successfully predict the susceptibility, morbidity, prognosis, and treatment response of KD. The susceptible gene(s) and mechanisms of immunopathogenesis for KD also need to be clarified. In our recently study, we uncovered KD patients showed remarkably hypomethylation at the gene promoters of toll like receptors (TLRs), especially TLR 1, 2, 4, 6, 8, and 9, and increased these TLR mRNA expressions [30]. Interestingly, TLR4 dependent macrophage signaling is associated with coronary arterial disease [31] and a TLR4 agonist has been shown to stimulate hepcidin expression [32]. Moreover, hepcidin not only controls iron metabolism but also plays a role in the pathogenesis of inflammation anemia [11]. Since anemia is often found in KD patients [19], we also indicated that higher hepcidin levels cause iron deficiency in the serum, thus reducing the availability of iron for erythropoiesis [20]. Furthermore, hepcidin associated iron homeostasis influences the ability of the macrophage polarization program [33] and aberrant activation and infiltration of macrophages is thought to be involved in the formation of arteritis in KD [34]. Moreover, we have shown in a recent study that high-dose aspirin significantly hinders the decrease of hepcidin levels and may be associated with a decrease in hemoglobin during the acute phase of KD [35].

Hepatocytes are the major producer of hepcidin. Since KD patients often present with hepatitis and jaundice [26], that may be one of the main reasons for modified hepcidin expression in KD patients. In fact, various studies have shown that higher AST and ALT levels and lower albumin levels were correlated with IVIG treatment resistance and CAL formation [27–29]. We further demonstrated that changes in hepcidin levels after patients received IVIG treatment were correlated with IVIG resistance and CAL formation [19]. In this study, we uncovered that plasma hepcidin was positively correlated with AST and ALT and negatively correlated with albumin prior to IVIG treatment in KD patients. In a recent study, plasma hepcidin was observed to increase significantly in ICU patients when compared with controls and was the highest in septic patients [36]. Hepcidin-induced low iron levels were also associated with both the short-term and long-term survival of critically ill patients [37]. Furthermore, Lin *et al*. identified hemoglobin as a useful marker for differentiating KD shock syndrome from toxic shock syndrome in a pediatric intensive care unit [38]. Therefore, the plasma hepcidin is greater in KD shock syndrome than in toxic shock syndrome. Therefore, the hepcidin and hemoglobin levels in KD can possibly be utilized clinically as a differential tool in the future. Based on the results in this study, we propose that hepcidin likely plays an important role in the pathogenesis and disease outcomes of KD patients. In the future, researcher should investigate the molecular machinery of hepcidin in KD and might unlock the mystery of KD.

However, this study still has limitations. The correlation coefficient between plasma hepcidin and several markers for KD severity were statistically significant but had values less than 0.4, thus potentially limiting the clinical application. Although *HAMP* demonstrated a borderline significance in the risk of KD in a Taiwanese population, clinical samples of a second population should be considered to confirm this significance.

In short, we reported herein that *HAMP* polymorphism might have a role in the disease susceptibility, as well as its expressions correlated with disease susceptibility, length of hospital stays, and albumin levels. This study provides a potential prognosis biomarker for Taiwanese children with KD.

## PATIENTS AND METHODS

### Patients

The Institutional Review Board of Chang Gung Memorial Hospital approved our study, and we obtained written informed consent from the parents or guardians of all the participating children. We recruited 381 KD patients (Table 1) for this study, and all of them were first treated with one dose of intravenous immunoglobulin (IVIG) (2 g/kg) over 12 h. We took two sets of blood samples: one within 24 h prior to IVIG treatment (pre-IVIG) and the other within 3 days following IVIG treatment (post-IVIG). We excluded any patients with symptoms that either did not correspond to the KD criteria or had experienced acute fever for less than 5 days. All patients were subjected to two-dimensional, pulse Doppler and color flow imaging at least three times within 8 weeks from the onset of the illness. If a patient was observed to have abnormal coronary arteries, an echocardiographic follow-up was scheduled every 3 to 6 months for one year and then once a year afterwards until the affected coronary arteries returned to normal, as reported in our previous studies [29, 39]. This current study carried out said the aforementioned echocardiography using a SONOS 5500 or 7500 cardiac scanner (Philips, Andover, MA, USA) and 5- to 8-MHz sector phased array transducers in order to visualize the diameters of both the right and left coronary arteries on the parasternal short-axis view of the aorta [4]. Pursuant to the guidelines of Japan's Ministry of Health, a CAL is considered a coronary artery if it has an internal diameter more than 3 mm (or 4 mm, if the patient was older than 5 years old) or if a segment has a diameter that is at least 1.5 times that of a contiguous segment, as observed on an echocardiogram. KD patients with coronary artery ectasia or dilatation that disappeared within the first 8 weeks after the onset of the illness were considered as having transient ectasia instead of CAL [40, 41]. We further classified coronary arteries based on whether aneurysms were present using the criteria published by the JCS Joint Working Group. A CAA (including medium and large aneurysms) was defined as a coronary artery with an internal diameter of at least 4 mm or, in children older than 5 years old, a segment with an internal diameter at least 1.5 times that of a contiguous segment as observed through echocardiography [40, 41]. A patient was considered to have responded to IVIG treatment if his/her fever abated 48 h after completing IVIG treatment and they had no recurrence of fever (defined as a temperature > 38°C) for at least 7 days afterwards, as well as visible improvement or normalization of inflammation [10, 29]. We also examined the plasma hepcidin in 85 patients with KD (Table 2) before and after they were treated with IVIG. The blood samples were immediately placed in tubes that already had heparin, while the other plasma aliquots were stored at –80°C until they were assayed.

### DNA extraction

We treated the obtained blood cells with a 0.5% SDS lysis buffer and then protease K (1 mg/ml) for 4 hours at 60°C to digest nuclear proteins. All DNA was extracted using a Gentra extraction kit, which was followed by 70% alcohol precipitation.

### Genotyping

We selected five tagging SNPs of *HAMP* (rs916145, rs10421768, rs3817623, rs7251432, and rs2293689) with a minimum allelic frequency of 1% in the Han Chinese population from the HapMap database (http://hapmap.ncbi.nlm.nih.gov/). All *HAMP* gene polymorphisms were found in the introns. We carried out genotyping using a TaqMan Allelic Discrimination Assay (Applied Biosystems, Foster City, CA, USA). PCR was performed quickly with a 96-well microplate in an ABI 9700 Thermal Cycler using the following thermal cycling conditions: denaturation at 95°C for 10 minutes, followed by 40 cycles of denaturation at 92°C for 15 seconds each, and then annealing and extension at 60°C for 1 min as previously described [42]. We used System SDS software version 1.2.3 to quantify and analyze fluorescence. The average genotyping success rate of our laboratory was 95.7%; therefore, the genotyping data of some subjects were unavailable. The genotype data of the 500 control subjects were obtained from Taiwan's Bio-Bank.

### Measurement of cytokines by enzyme-linked immunoassay (ELISA)

The ELISA kits that we used for plasma hepcidin-25 were commercially available competitive assays with synthetic hepcidin (Bachem Biosciences, St. Helens, United Kingdom, Catalog Number: S-1337), and we used the performance protocol described in one of our previous studies [19].

### Statistical analysis

All data in this study are presented as mean ± standard error. The genotypes and allele frequencies that correlated with KD susceptibility and disease outcomes (CAL formation, IVIG treatment response, and aneurysm) were analyzed using the Chi-square test, which was then used with 1 degree of freedom to carry out the Hardy-Weinberg equilibrium. Changes between the values before and after IVIG treatment were evaluated with a paired-sample *t-test*. Two-sided *p*-values < 0.05 were considered statistically significant. All statistical analyses were performed using SPSS version 22.0 for Windows (SPSS software, Inc., Chicago, IL, USA) and JMP 9.0 for Windows.

## SUPPLEMENTARY MATERIALS TABLE



## Abbreviations

CAA: coronary artery aneurysms
CAL: coronary artery lesions
IVIG: intravenous immunoglobulin
KD: Kawasaki disease
SN*P*: single nucleotide polymorphism.
