# Supplementary material for: Correlation of HAMP gene polymorphisms and expression with the susceptibility and length of hospital stays in Taiwanese children with Kawasaki disease
Source: Oncotarget. 2017 May 8;8(31):51859–68. doi: 10.18632/oncotarget.17700 (PMC5584296; doi:10.18632/oncotarget.17700)
Supplement: Supplementary file 1 [file oncotarget-08-51859-s001.pdf]

Correlation of *HAMP* gene polymorphisms and expression with the susceptibility and length of hospital stays in taiwanese children with kawasaki disease

Supplementary Materials

Supplementary Table 1: Basic characteristics of the tag single-nucleotide polymorphisms (SNPs) in *HAMP*

Position on Chromosome 19 (hg38)	Variant	Ref	Alt	Allele frequency*					HWE
				AFR	AMR	ASN	EUR	TWB	(*p* value)
35767884	rs916145	C	G	0.06	0.05	0.35	0.12	0.35	0.837
35772899	rs10421768	A	G	0.19	0.18	0.01	0.23	0.01	0.857
35773146	rs3817623	G	T	0.00	0.00	0.06	0.00	0.06	0.674
35775441	rs7251432	A	G	0.25	0.48	0.37	0.48	0.38	0.172
35775626	rs2293689	C	T	0.05	0.03	0.09	0.01	0.10	0.571

Ref: reference allele, Alt: Alternative allele. *Frequency of the Alt allele. AFR: African. AMR: Ad Mixed American. ASN: Asian. EUR: European. TWB: the Taiwan Biobank. HWE: Hardy-Weinberg equilibrium.
